# Prior knee arthroscopy effects on subsequent total knee arthroplasty: A protocol of match-controlled study

**DOI:** 10.1097/MD.0000000000019844

**Published:** 2020-04-24

**Authors:** Feng Hu, Xulin Chen, Yingjie Wu, Wei Liu

**Affiliations:** a Department of Orthopaedics, the Second Affiliated Hospital of Guangxi Medical University; b Department of Plastic and Aesthetic Surgery, the First Affiliated Hospital of Guangxi Medical University, Guangxi, China.

**Keywords:** complication, prior knee arthroscopy, protocol, revision, total knee arthroplasty

## Abstract

**Background:**

Currently, there exists a paucity of literature about the impact of prior knee arthroscopy on subsequent total knee arthroplasty (TKA). The purpose of this study was to compare outcomes of patients undergoing TKA after prior knee arthroscopy with a matched cohort of control subjects with primary osteoarthritis and no history of arthroscopy.

**Methods:**

We reviewed patients who underwent primary TKA at our academic center from January 2011 to December 2017. Of these, we included 68 patients (70 knees) that were performed TKA following knee arthroscopy. The groups were split by sex, age to within 6 years, and body mass index within 5 kg/m^2^. A 1:2 matching algorithm was applied. Outcome measures included surgical time, intraoperative estimated blood loss, Oxford Knee Score, range of movement, complications, and revision rate.

**Results:**

This study had limited inclusion and exclusion criteria and a well-controlled intervention.

**Conclusion:**

This clinical trial is expected to determine whether prior knee arthroscopy is associated with reduced functional outcomes or increased risks of revision and complications following TKA.

**Trial registration:**

This study protocol was registered in Research Registry (researchregistry5413).

## 1 Introduction

Knee osteoarthritis is a common condition that has necessitated the use of treatments ranging from conservative measures such as oral medications and intra-articular injection to surgery. Over the past decade, the use of knee arthroscopy for treatment of various arthropathy has grown rapidly. As a reproducible and minimally invasive method, there is currently no conclusive evidence in the literature that arthroscopy cures or arrests osteoarthritis.^[1,2]^ Knee osteoarthritis can progress and eventually require total knee arthroplasty (TKA) for end-stage disease in spite of arthroscopic intervention. Although the effect of knee arthroscopy in the management of the degenerate knee arthritis remains a contentious issue, it is estimated that >980,000 knee arthroscopies and 630,000 primary TKAs are performed annually in the United States.^[3,4]^ Johanson et al^[1]^ estimated that 10.2% of patients over the age of 65 who underwent arthroscopy for osteoarthritis required a TKA within 1 year, and 32.5% underwent a TKA within 9 years post-arthroscopy. Therefore, the imminent demand for good postoperative outcomes reinforces the importance of determining the link between prior arthroscopy and future TKA.

Currently, there remains a paucity of literature about the impact of prior knee arthroscopy on subsequent TKA. Piedade et al^[5]^ examined a group of 60 cases who underwent primary TKA following knee arthroscopic debridement and compared them with a cohort of 1119 TKAs who had no previous surgery. The authors found that patients with prior arthroscopic surgery were associated with higher rate of postoperative complications and failures. Subsequent studies by Werner et al^[6]^ and Barton et al^[7]^ also showed significantly higher rates of postoperative complications among patients undergoing TKA within 6 months after ipsilateral knee arthroscopy when compared with control groups. However, other studies found no clinical or functional outcome differences between the 2 cohorts.^[8,9]^

Given that current literature has not been conclusive and there are limited studies evaluating or discussing this topic, the purpose of this study was to compare outcomes of patients undergoing TKA after prior knee arthroscopy with a matched cohort of control subjects with primary osteoarthritis and no history of arthroscopy. We hypothesized that patients with prior knee arthroscopy would demonstrate similar functional outcomes, complication rates, and revision rates after subsequent primary knee arthroplasty when compared with a control group.

## 2 Materials and methods

This study will be performed and reported in accordance with the Strengthening the Reporting of Observational studies in Epidemiology checklist.

### 2.1 Patients

This retrospective matched cohort study was approved by the institutional review board in our hospital (PE2020039) and was registered in the research registry (researchregistry5413). Selection criteria were set as follows: patients that were over 18 years old and could cooperate with us for treatment and postoperative observation; all patients had undergone primary TKA during hospitalization; patients in the intervention group did not undergo open knee procedures or any arthroscopic ligament reconstruction; patients were not associated with other diseases that may have an influence on knee motion after surgery; follow up >1 year; full demographic and follow-up data. We reviewed patients who underwent primary TKA at our academic center from January 2011 to December 2017. Of these, we included 68 patients (70 knees) that were performed following knee arthroscopy (arthroscopy group). To reduce the effect of selection bias and potential confounding, these patients were then matched with 1 separate cohort of patients who had not undergone arthroscopy surgery (control group). The groups were matched for sex, age to within 6 years, and body mass index (BMI) within 5 kg/m^2^. A 1:2 matching algorithm was applied.

### 2.2 Surgical details

All TKAs and prior arthroscopies in this study were carried out by either the senior author, or by fellows under his direct supervision. Knee arthroscopy was performed after failure of conservative measures such as rest, physical therapy, anti-inflammatory medication, or intra-articular injection. In terms of the TKA, a tourniquet was used in all cases, general anesthesia was administered to each patient before incision, and the operative knee was prepared and draped in a conventional sterile fashion. It was performed through a midline skin incision and a medial parapatellar approach. The femoral component rotation was set at 3° of external rotation relative to the posterior condylar axis. After making bone cuts based on anatomic landmarks, judicious soft tissue releases were conducted to create a balanced knee in flexion and extension. Patients in both cohorts received identical fixed-bearing, posterior-stabilized TKA (Waldemar Link GMBH and Co, Hamburg, Germany) with cemented fixation. After final relocation, the surgical wound was irrigated with sterile solution, the capsule was repaired, and a drain was placed. The wound was closed and dressed, and the patient was safely awakened.

### 2.3 Postoperative care

Postoperative drainage lasts 1 to 2 days until flow volume is <30 mL. All patients received the same standardized postoperative multimodal pain protocol, with 4 doses of 1 g of acetaminophen, 2 doses of celecoxib 200 mg, and morphine (first 48 hours) or tramadol (after 48 hours) for pain exacerbations. All patients underwent the same postoperative rehabilitation program, with partial weight bearing with the use of crutches for the first postoperative day and active range of movement (ROM) exercises.

### 2.4 Outcome evaluation

Patient demographic variables including sex, age, surgical side, and BMI were recorded. Outcome measures included surgical time, intraoperative estimated blood loss, Oxford Knee Score (OKS), ROM, complications, and revision rate. Surgical time and estimated blood loss were obtained from our hospital database, as well as electronic and paper records. The OKS and ROM were obtained both before and after surgery at a minimum of 1 year postoperatively. For patients who were not seen recently, the scores were obtained via telephone. Postoperative complications and revision procedures were documented during routine collection of follow-up data. All data were independently verified by a detailed review of hospital operative reports, anesthesia records, and clinical records. Data were abstracted by 1 of 2 research personnel blinded to patient group and study aim. A minimum of 1-year follow-up was utilized for this study with a mean follow-up of 3.2 years. Follow-up duration was determined by last patient contact via office visit or telephone update.

### 2.5 Statistical analysis

SPSS Statistics for Windows, version 20.0 (IBM Corporation, Armonk, NY) statistical software was used to analyze the measurement results. Chi-squared test was used for comparison of sex, surgical side, complications, and revisions. The *F* test was used to determine if continuous data demonstrated equal variances. The paired or unpaired 2-tailed *t* tests were performed to compare continuous data sets regarding surgical time, estimated blood loss, OKS, and ROM, with statistical significance set at *P* ≤ .05. Descriptive statistics were calculated, including means, standard deviations, and proportions.

## 3 Result

The results will be shown in Tables 1–5.

**Table 1:**
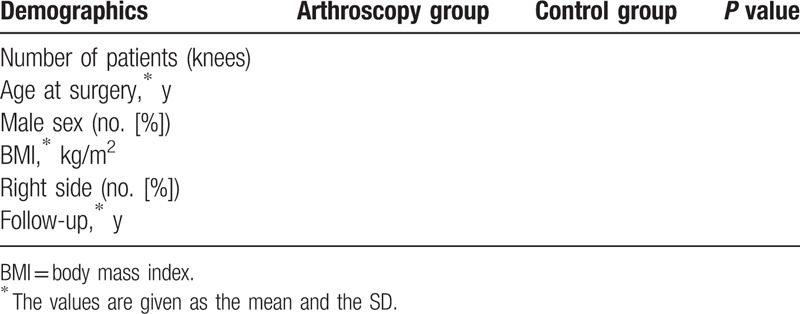
Patient baseline demographics.

**Table 2:**
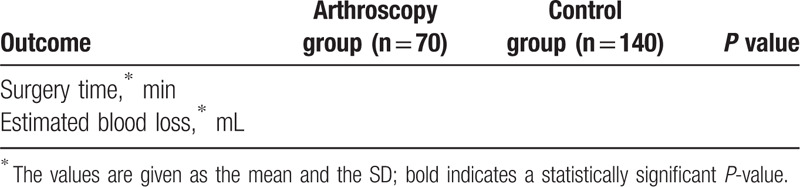
Intraoperative outcomes.

**Table 3:**
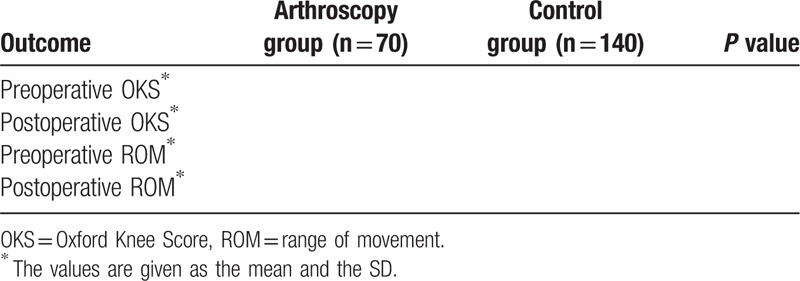
Functional outcomes measured preoperatively and at latest follow-up.

**Table 4:**
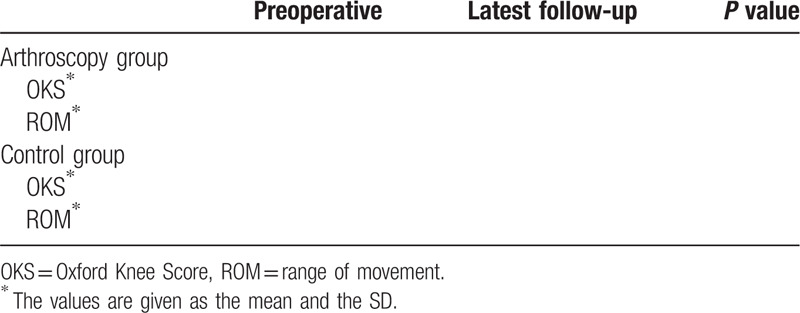
Improvements from preoperatively to latest follow-up.

**Table 5:**
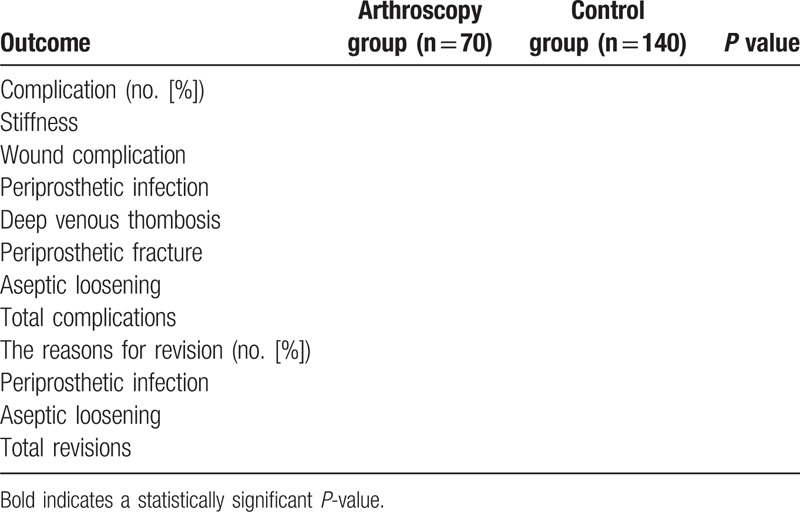
The adverse outcomes in the 2 groups.

## 4 Discussion

As the field of knee arthroscopy grows, it is likely that an increased proportion of patients undergoing TKA will have a history of arthroscopic surgery on their operative knee. There is little information about the impact of arthroscopy surgery on the outcome of subsequent TKA. The aim of this study is to determine whether prior knee arthroscopy is associated with reduced functional outcomes or increased risks of revision and complications following TKA.

Prior knee arthroscopy may lead to intra-articular adhesions, soft-tissue scarring, and cartilage degeneration, thereby possibly resulting in impaired functional scores.^[10–14]^ Functional results from prior studies evaluating TKA in the setting of prior knee arthroscopy are conflicting, with some studies showing worse scores in arthroscopy group^[7]^ and others finding no difference in outcomes.^[5,8]^ Three prior studies have reported an increase in postoperative complications and revision rates following TKA in patients who had undergone previous knee arthroscopy.^[15–17]^ A retrospective study using the PearlDiver Patient Records Database from 2008 to 2011 showed that the incidences of infection, stiffness, and venous thromboembolism were higher in patients who underwent TKA within 6 months after knee arthroscopy compared with controls. However, there was no increase in complication rates when TKA was performed >6 months after knee arthroscopy. The authors hypothesized that the increased infection risk may be a result of the capsular violation from recent arthroscopy or persistent synovitis in the knee.^[6]^ Piedade et al^[5]^ performed a retrospective non-matched study of 60 patients who underwent TKA after knee arthroscopy with mean age at the time of TKA of 69 years and minimum follow-up period of 2 years (mean of 3.6 years). There were higher rates of stiffness and aseptic loosening as well as a worse survival curve in those who underwent knee arthroscopy surgery before TKA compared with controls. Barton et al^[7]^ also performed a retrospective study of 186 patients who underwent TKA after anterior cruciate ligament reconstruction, of mean age of 65 years and minimum of 3-month follow-up (mean of 3.4 years). The authors found that patients who underwent TKA within 6 months led to an increased rate of revision surgery. However, these studies were non-matched and unadjusted for many confounding variables.

The limitations of our study included those inherent in any retrospective cohort study, including the possibility of selection or observational bias. This study also did not address long-term follow-up (10 years) as our study relied on electronic medical records kept since 2011. The authors recognize that longer term follow-up is critical in determining the influence of prior knee arthroscopy on TKA specifically on infection, implant loosening, revision, and long-term function outcomes. Additionally, although we performed a matched study based on sex, age, and BMI, it is likely that there were other preoperative features that we could have controlled for that may have led to alternative results. Although length of time between arthroscopy and arthroplasty has been previously suggested to play a role in complication or revision rates, there was no sufficient data to perform further subgroup analyses in our study.

## Author contributions

Feng Hu planned the study design and wrote the study protocol. Xulin Chen and Yingjie Wu reviewed the study protocol. Feng Hu, Xulin Chen, Wei Liu, and Yingjie Wu will recruit participants and collect data. Feng Hu wrote the manuscript. All of the authors have read, commented on, and contributed to the submitted manuscript.

Wei Liu orcid: 0000-0003-3321-4403.
